# Continuous ambulatory peritoneal dialysis catheter placement: Is omentectomy necessary?

**DOI:** 10.4103/0974-7796.68858

**Published:** 2010

**Authors:** Joseph P. Kavalakkat, Santosh Kumar, Karthikeyan Aswathaman, Nitin S. Kekre

**Affiliations:** Department of Urology, CMC, Vellore, Tamil Nadu, India

**Keywords:** Chronic renal failure, dialysis, omentectomy

## Abstract

**Context:**

There are different methods of continuous ambulatory peritoneal dialysis (CAPD) catheter placement. Open surgical technique is a widely followed method. The complication rate following catheter placement varies and catheter blockage due to omental plugging is one of the main reasons.

**Aim:**

To analyze the need for routine omentectomy during CAPD catheter placement.

**Materials and Methods:**

This was a retrospective analysis of 58 CAPD catheter placements performed between July 2002 and June 2007. Tenckhoff double cuffed catheter was used in all. The postoperative complications were analyzed.

**Results:**

There were 44 males and 14 females. The mean age was 51 years ranging from 15 to 76 years. Of these, 40 (69%) patients underwent omentectomy (group A) and 18 (31%) did not (group B). Laparoscopic and open techniques were performed in 5 and 53 patients, respectively. Omentectomy was not performed in 13 patients with open technique and all the five in the laparoscopic group. One patient in group A developed hemoperitoneum which was treated conservatively. None from group A developed catheter blockage, whereas five (27.8%) from group B developed catheter blockage postoperatively. The median time interval between the primary procedure and development of catheter blockage was 45 days (ranged from 14 to 150 days).

**Conclusions:**

Omentectomy during CAPD catheter placement prevents catheter blockage and secondary interventions.

## INTRODUCTION

Continuous ambulatory peritoneal dialysis (CAPD) is being widely used as an alternative to hemodialysis, after its inception by Papovich *et al*. in 1976.[1] Even though peritoneal dialysis provides many advantages compared to hemodialysis, the incidence of complications like peritonitis and catheter malfunction can be as high as 70%.[2] The advantages of peritoneal dialysis include more liberal dietary intake of protein, potassium, sodium and fluids, elimination of need for anticoagulation, increased patient mobility and lower cost.[3] Hematocrit levels are often higher than for patients receiving hemodialysis, and gradual and continuous ultrafiltration may provide better blood pressure control. Because it is a form of self-care, peritoneal dialysis promotes patient independence.

The intrinsic properties of the greater omentum are such that if it comes into contact with a CAPD catheter, it will wrap around the catheter in an attempt to isolate this foreign body from the rest of the peritoneal cavity. The omentum then partially or totally occludes the lumen of the CAPD catheter. Other causes of catheter malfunction include kinking and malposition of tip. Of these, omental wrapping is the most common cause of mechanical malfunction. It can be either spontaneous or secondary to peritonitis. One simple method to avoid omentum-related complications is to perform a partial omentectomy at the time of catheter insertion.

Studies have suggested that omentectomy at the time of catheter placement increases catheter survival by preventing obstruction.[4] Other methods used to salvage nonfunctioning catheters, like omentopexy and catheter repositioning, have higher recurrence rates of obstruction.[5] There are no randomized studies comparing the outcome of omentectomy versus no omentectomy. This study aims to analyze whether omentectomy is required during CAPD catheter placement.

## MATERIALS AND METHODS

Hospital records of all patients who underwent CAPD catheter placement from July 2002 to June 2007 were retrospectively analyzed. Majority of the procedures were done under general anesthesia. The peritoneal cavity was opened and patient tilted head down, allowing the bowel to fall away from the pelvis. Under direct vision, the Tenckhoff catheter was placed into the recto-vesical or recto-uterine pouch. Partial omentectomy was done as per the surgeon’s discretion at the time of operation. Double cuffed Tenckhoff catheter was used for all patients. The inner dacron cuff was secured to the parietal peritoneum. The catheter was then tunneled subcutaneously through a separate exit site. In the operating room, dialysate exchange was performed using a 1-l bag to ensure a water tight seal of the peritoneum. All intraoperative and postoperative complications including subsequent admissions were noted. Student’s *t* test and chi square test were used for statistical analysis.

## RESULTS

Of the 58 patients who underwent CAPD catheter placement, 44 (75%) were males and 14 (25%) were females. The mean age was 51 years (ranged from 15 to 76 years). Open catheter placement was done in 53 patients and 5 patients underwent laparoscopic catheter placement. Partial omentectomy was done in 40 patients (group A) who had an open technique and it was not done in the rest 18 (group B). The mean operating time in those who underwent omentectomy along with CAPD catheter insertion was 90±17.3 minutes, whereas those without omentectomy had a mean operating time of 80±10.5 minutes (*P* value 0.0274) which was statistically significant.

The decision to do omentectomy was at surgeon’s discretion depending on whether he could access the omentum from the incision, the size of the omentum and whether it could be pulled near the field of the catheter to obstruct it. Of the 40 who underwent omentectomy, none developed catheter blockage, whereas five (27.8%) in group B developed catheter block (*P* value 0.0005). All of them underwent re-exploration and omentum was found to be wrapped around the catheter and omentectomy was performed. One patient had to undergo two interventions. During the first re-exploration, the catheter tip had migrated which was repositioned. Four weeks later, he again developed catheter blockage. On exploration, the catheter was found to be blocked by the wrapped omentum requiring partial omentectomy. During the follow-up period, none of the re-explored patients had catheter-related complications. One from group A developed hemoperitoneum which was managed conservatively. Five (12.5%) from group A and two (11.1%) from group B developed pericatheter leak (*P* value 0.879) which was statistically not significant. All these were treated conservatively.

## DISCUSSION

In our series of the 58 patients who underwent CAPD catheter placement, partial omentectomy was done in 40. Of these 40 who underwent partial omentectomy, none developed catheter blockage, whereas 5 out of 18 who did not have partial omentectomy (27.8%) developed catheter block. All of them underwent re-exploration and omentum was found to be wrapped around the catheter and partial omentectomy was performed. During the follow-up period, none of the re-explored patients had catheter-related complications.

The incidence of CAPD catheter obstruction varies from 2 to 32% in different studies. One major factor which influences this is whether partial omentectomy was done or not during the primary surgery. Early failure rate, that is, within 4 weeks of catheter placement, is usually associated with obstruction rather than peritonitis.[4] The obstruction is usually caused by omentum wrapping around the catheter and occluding the ports.[6] During the catheter insertion, the tip is placed deep into the pelvis to avoid the catheter being obstructed by the omentum. However, the peritoneal catheters are frequently blocked. Routine partial omentectomy at the time of catheter placement may help to prevent this complication.

Reissman *et al*. reported only 2% incidence of catheter obstruction in a series of 60 consecutive patients who underwent routine omentectomy during CAPD catheter placement.[7] Sanderson *et al*. showed 60% reduction in catheter obstruction due to routine omentectomy in a series of 260 CAPD catheter placement operations. One year catheter survival was 90%.[8] Nicholson *et al*. performed omentectomy in 113 cases in a series of 300 consecutive CAPD catheter placements and observed a significant improvement in catheter survival (78% of catheter survival in the omentectomy group compared to 50% in the no-omentectomy group during 5 years of follow up) in those who had undergone partial omentectomy.[9] In our series, none of the patients in the omentectomy group developed obstruction of the catheter.

At the same time, five patients (27.8%) in our series who did not undergo partial omentectomy developed catheter block due to wrapped omentum, which is in accordance with the earlier reports in the literature. In a series by Stefano *et al*., the most frequent cause of peritoneal dialysis catheter malfunction necessitating intervention was omental wrapping. The incidence of this complication in that series was as high as 32%. But Davis *et al*. reported that more than 60% needed revision because of mechanical obstruction in the long course.[10] Laparoscopic procedures limited to liberate the catheter from omental tissue are associated with a high rate of recurrent obstruction. This points to the need for preventive measures like omentopexy or omental epiplopexy, apart from omentectomy.[11]

One case of hemoperitoneum was reported in a series of 300 CAPD catheter placements by Nicholson *et al*., in which 113 underwent omentectomy. Abdomen was re-explored and found to have a slipped ligature. However, the catheter was salvaged. In our series also, there was one case of hemoperitoneum which was treated conservatively. Another issue regarding omentectomy is whether it will add much to the operating time or not. The mean operating time in those who underwent omentectomy along with CAPD catheter insertion was 90±17.3 minutes, whereas those without omentectomy had a mean operating time of 80±10.5 minutes.

## CONCLUSION

Our results demonstrate that partial omentectomy was an independent factor associated with improved CAPD catheter survival. Partial omentectomy prevents the mechanical complications following CAPD catheter placement. It is an easy procedure and does not increase the operating time. But prospective randomized studies are needed before recommending omentectomy.
